# The Accurate and Exclusive Quantification of Somatic Cells in Raw Milk with an OPD-Cu^2+^ System-Based Colorimetric Method

**DOI:** 10.3390/foods13182890

**Published:** 2024-09-12

**Authors:** Menghui Xie, Meng Wang, Siyuan Liu, Yingying Liu, Ziquan Wang, Guoping Zhou, Zhiwei Sui

**Affiliations:** 1 Center for Advanced Measurement Science, National Institute of Metrology, Beijing 100029, China; xiemenghui2021@163.com (M.X.); mengwang@nim.ac.cn (M.W.); liusy@nim.ac.cn (S.L.); liuyy@nim.ac.cn (Y.L.); wangzq@nim.ac.cn (Z.W.); 2 School of Life Science and Technology, Wuhan Polytechnic University, Wuhan 430023, China; wjczgp@163.com

**Keywords:** somatic cell count, raw milk, OPD, Cu^2+^, colorimetric assay

## Abstract

The somatic cell count (SCC) refers to the number of somatic cells present in each milliliter of raw milk and serves as a crucial indicator of dairy cow udder health and raw milk quality. Traditional SCC detection methods are often time-consuming, expensive, and susceptible to bacterial interference, rendering them unsuitable for the rapid and unbiased assessment of raw milk quality. Consequently, there is an urgent need for a low-cost, accurate, and user-friendly SCC quantification method. Here, a method based on an OPD-Cu^2+^ system for SCC quantification was developed. It was found that OPD oxidation signals exhibited a linear correlation with SCC. Following optimization, the detection system was established with a Cu^2+^ concentration of 25 μM, an OPD concentration of 2 mM, and an incubation time of 15 min. Furthermore, the method demonstrated significant resistance to bacterial interference, though it produced weaker signals in response to bacteria. The somatic cell recovery rate in milk after pretreatment was 88.9%, and SCC was quantified accurately within 45 min, with a linear range of 10^4^–10^6^ cells/mL. In summary, the method developed is cost-effective, straightforward, and facilitates precise somatic cell quantification, offering significant practical value and a new approach for SCC detection in raw milk.

## 1. Introduction

The somatic cell count (SCC) represents the total number of somatic cells in each milliliter of raw milk. Somatic cells in raw milk are composed of 98–99% macrophages, lymphocytes, polymorphonuclear neutrophilic leukocytes, and 1–2% mammary epithelial cells [1]. The SCC is a key indicator for assessing udder health and raw milk quality in dairy cows. External influences, particularly harmful bacteria that invade the udder, activate numerous immune cells. These cells migrate into the udder via the bloodstream, causing tissue damage and inflammation. Subsequently, these immune cells are secreted with milk, leading to an increased SCC [2,3,4]. Typically, the SCC in raw milk ranges from about 2 × 10^4^ cells/mL to 4 × 10^5^ cells/mL. An SCC exceeding 5 × 10^5^ cells/mL indicates that the cow is likely suffering from mastitis [5]. Milk from affected cows tends to be lower in volume, nutrient content, and may contain multiple toxins, potentially causing severe food poisoning symptoms even after pasteurization [6,7]. Thus, precise detection of somatic cells is crucial for monitoring udder health, ensuring product quality, and safeguarding consumer health.

While the microscopic method remains the primary regulatory approach for quantifying SCC [8], it is complex to perform and prone to detection bias due to visual fatigue [9]. To overcome these limitations, various alternative techniques for rapid SCC detection have been developed. As outlined in Table 1, somatic cell detection methods can be broadly divided into two categories: direct detection methods, including fluorescence photoelectric counting [10], Coulter counting [11], and flow cytometry [12], and indirect detection methods, such as the California mastitis test (CMT) [13], ATP bioluminescence detection [14], and conductivity and pH detection [15]. Direct detection methods, while effective, are costly and require specialized operators, limiting their widespread use [16]. Indirect detection methods often lack sufficient sensitivity and specificity for accurate SCC counting [17], as they cannot fully exclude bacterial influence on test signals. For instance, Coulter counting, though rapid, does not specifically identify DNA from somatic cells, leading to interference from bacterial cells in milk samples and higher count analysis [18]. Additionally, the CMT only provides positive or negative results for mastitis without identifying the specific pathogen [19], complicating the exclusion of bacterial interference. Therefore, a low-cost, user-friendly, and accurate somatic cell quantification method with strong anti-interference capability is urgently needed.

O-phenylenediamine (OPD) can be oxidized to OPDox (2,3-diaminophenazine) by certain metal ions, including Cu^2+^, Ag^+^, or hydrogen peroxide (H_2_O_2_) [20,21,22]. The resulting OPDox exhibits a visible pale-yellow color, making it suitable for colorimetric sensing systems. For instance, Yang et al. have developed a dual-signal sensing strategy based on ratiometric fluorescence and colorimetry to determine Cu^2+^ and glyphosate, utilizing the oxidative ability of Cu^2+^ towards OPD [23]. Deng et al. developed a label-free biosensor based on the OPD-Cu^2+^ system for selective detection of gram-negative bacteria [24]. Wang et al. created a colorimetric and fluorescent dual-channel sensing platform for rapid detection of *Escherichia coli* using the OPD-Cu^2+^ system [25]. Although the OPD-Cu^2+^ system-based colorimetric method shows promise for bacterial cell quantification, its efficacy in accurately quantifying somatic cells in raw milk remains to be determined.

The objective of this study was to develop a precise quantification method with strong anti-interference capability for detecting the SCC in raw milk. Specifically, the study focused on the accurate quantification of the SCC in raw milk using the OPD-Cu^2+^ system. In this method, somatic cells effectively consumed Cu^2+^, thereby inhibiting the Cu^2+^-induced oxidation of OPD to OPDox, resulting in a significant reduction in OPDox signals. The assay parameters were optimized, and the recovery and detection range of the method were evaluated. Additionally, the method exhibited no interference from bacterial cells, enabling the cost-effective, simple, and accurate quantification of somatic cells. This detection method holds significant potential for enhancing the reliability of mastitis diagnosis in dairy cows.

## 2. Materials and Methods

### 2.1. Cell Strains, Bacterial Strains, and Culture Conditions

Somatic cells (HEK293T, ATCC CRL-3216^TM^), *Pseudomonas fluorescens* (*P. fluorescens*, CICC 21620), *Acinetobacter baumannii* (*A. baumannii*, ATCC 17978), and *Lactococcus lactis* (*L. lactis*, ATCC 19257) were utilized in this study. These cells and bacterial strains were also employed to assess the anti-interference capability of the method.

The frozen cell samples were thawed using a 37 °C water bath. Following thawing and centrifugation (100× *g*, 5 min), the cell pellet was resuspended in Dulbecco’s modified Eagle’s medium (DMEM, Thermo Fisher Scientific, Waltham, MA, USA) supplemented with calf serum (10%, Thermo Fisher Scientific). The cells were plated into culture dishes with 10 mL of DMEM and incubated at 37 °C in a CO_2_ incubator (5%, Thermo Fisher Scientific). The culture medium was refreshed the following day [26]. After 2 days of culture, 293T cells were harvested. To minimize the impact of the medium color on the reaction system, the cells were washed twice at 100× *g* for 10 min. The pellet was then resuspended in 10 mM Tris-HCl (pH 7.5; Solarbio, Beijing, China), and the cell concentration was measured by flow cytometry for further analysis.

The bacterial strains utilized in the experiments were activated on nutrient agar (NA, Land Bridge, Beijing, China) plates. *P. fluorescens* and *L. lactis* were cultured at 30 ± 0.5 °C, whereas *A. baumannii* was incubated at 37 ± 0.5 °C. Individual colonies were selected and transferred to a nutrient broth (NB, Land Bridge) for oscillatory incubation [27,28]. Bacteria in the logarithmic growth phase were then harvested for further analysis. The bacterial pellet was collected (5000× *g*, 5 min) and promptly resuspended in 1 mL of Tris-HCl. A bacterial suspension with a concentration of about 10^8^ CFU/mL was prepared by mixing the three bacterial strains in a 1:1:1 ratio for subsequent examination.

### 2.2. Feasibility of the OPD-Cu^2+^ System for the Detection of Somatic Cells

**Figure 1:**
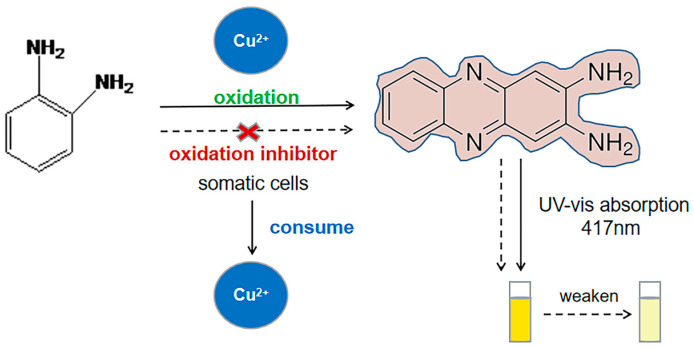
Schematic diagram of somatic cell detection by the OPD-Cu^2+^ system-based colorimetric method. The somatic cells effectively consumed Cu^2+^, inhibiting the Cu^2+^-triggered oxidation of OPD to OPDox. Consequently, the OPDox signals significantly decreased.

To determine if the OPD-Cu^2+^ system reacted with somatic cells (Figure 1), four experimental setups were prepared. The compositions of the Tris-HCl buffer for the four systems were as follows: (1) OPD alone; (2) OPD with a Cu^2+^ solution (prepared from CuSO_4_•5H_2_O, ready-to-use); (3) OPD with a somatic cell solution; and (4) an OPD, Cu^2+^, and somatic cell solution. The mixtures were incubated at 37 °C for 2 h, then allowed to cool to room temperature. The reaction mixtures were centrifuged (10,000× *g*, 5 min), and the absorbance of the supernatants was measured using an ultraviolet-visible spectrophotometer (SPECTROstar^®^ Nano, BMG LABTECH, Offenburg, Germany) at 417 nm [29].

### 2.3. Optimization of the OPD-Cu^2+^ System Detection Conditions

#### 2.3.1. Optimization of the Cu^2+^ Concentration

Various concentrations of Cu^2+^ solution, ranging from 0 μM to 50 μM, were combined with either 2 × 10^5^ cells/mL of cell mixture or 2 × 10^7^ CFU/mL of bacterial mixture. Subsequently, 40 μL of OPD solution (20 mM) was added, resulting in a final reaction volume of 400 μL. The solutions were incubated at 37 °C for 2 h and then allowed to cool to room temperature [30]. To minimize the impact of solution turbidity on the results, the samples were centrifuged at 10,000× *g* for 5 min [31]. The supernatant was then collected for UV absorption measurement. The signals from the cell and bacterial solutions were compared, and the optimal Cu^2+^ concentration in the reaction system was assessed based on the recorded data.

#### 2.3.2. Optimization of the OPD Concentration

The somatic cell mixture was combined with Cu^2+^ (25 μM) in Tris-HCl buffer. Various concentrations of OPD solution were subsequently introduced and thoroughly mixed, resulting in a final reaction volume of 400 μL, with OPD concentrations ranging from 0 mM to 5 mM. The mixtures were incubated at 37 °C for 2 h, followed by cooling to room temperature for UV absorbance measurements. Changes in the UV absorption of the supernatant were recorded. The optimal OPD concentration in the reaction system was determined based on these recorded results.

#### 2.3.3. Optimization of the Incubation Time

Somatic cells at concentrations varying from 10^4^ to 10^6^ cells/mL were combined with Cu^2+^ (25 μM). The OPD solution was added to a final concentration of 2 mM, resulting in a total reaction volume of 400 μL. The mixture was incubated at 37 °C, with UV absorbance measured at 15-min intervals. The optimal incubation time for the reaction system was established based on these recorded results.

### 2.4. Somatic Cell Detection with the Developed Method

The cell suspension was diluted with Tris-HCl buffer to achieve cell concentrations of 10^4^ to 10^6^ cells/mL. The samples were combined with the optimized concentrations of Cu^2+^ and OPD, and then incubated with shaking at 37 °C for 15 min. Following incubation, the mixture was cooled to room temperature for 5 min before UV absorption was measured. The detection range of the method was determined and the limit of detection was calculated based on these results.

To investigate the potential mechanism of the OPD-Cu^2+^ detection system, two additional experiments were conducted: (1) carbonyl cyanide 3-chlorophenylhydrazone (CCCP, Thermo Fisher Scientific) was added to the OPD-Cu^2+^-somatic cell solution at a concentration of 50 μM to inhibit the cellular redox reaction, and (2) negatively charged magnetic beads at a concentration of 1 mg/mL were introduced to simulate the cell surface charge. The mixture was incubated at 37 °C for 2 h, then cooled to room temperature before the absorbance value of the supernatant was measured immediately.

### 2.5. FCM Analysis

FCM analysis was performed using a volume-based flow cytometer equipped with 488 nm and 638 nm lasers, each with a power of 20 mW (A50-Micro model, Apogee, Hemel Hempstead, UK) [32]. A 500 µL volume of the cell suspension was used for sampling. To this, 1 µL of propidium iodide (PI) and 1 µL of SYTO 9 were added and thoroughly mixed. The solution was protected from light for 15 min prior to assay. Fluorescence emissions from SYTO 9 were captured in the green fluorescence (FL) channel FL1 (525 nm), while emissions from PI were captured in the red fluorescence channel FL2 (680 nm). The sample flow rate was adjusted to 10.5 μL/min, and detection voltages for the side scatter, FL1, and FL2 were set at 200 V, 265 V, and 450 V, respectively. PBS was used as the sheath fluid. Events were analyzed by FCM for 30 s, with each sample being evaluated in triplicate.

### 2.6. Evaluation of the Bacterial Interference with the Somatic Cell Detection

The resulting cell suspension was diluted with Tris-HCl buffer to achieve cell concentrations of 2 × 10^4^, 5 × 10^4^, 2 × 10^5^, 4 × 10^5^, 6 × 10^5^, and 2 × 10^6^ cells/mL. Each sample was combined with 2 × 10^6^ CFU/mL bacteria and the optimal concentration of Cu^2+^ and OPD solution. The mixture was then incubated with shaking at 37 °C for 15 min. After incubation, the mixture was cooled, and the supernatant was collected for UV absorbance measurement. UV absorption was recorded at various concentrations to assess the anti-interference capability of the OPD-Cu^2+^ system.

### 2.7. Bacteria Detection with the Developed Method

The bacterial suspension prepared in Section 2.1 was diluted with Tris-HCl buffer to achieve bacterial concentrations of 2 × 10^4^, 2 × 10^5^, 2 × 10^6^, 2 × 10^7^, and 2 × 10^8^ cells/mL. The samples were then combined with Cu^2+^ and OPD solutions and incubated with shaking at 37 °C for 2 h. Following incubation, the mixture was cooled to room temperature, and the UV absorption of the different concentrations of the cell suspension was recorded immediately.

### 2.8. Preparation and Pretreatment of the Artificially Contaminated Milk Samples

Artificially contaminated samples were created by introducing various concentrations of somatic cells into commercially sterilized milk, with cell concentrations in the samples ranging from 10^4^ cells/mL to 10^6^ cells/mL. Tris-HCl buffer was added to the milk sample as a negative control. Commercial milk samples (Mengniu Dairy, Hohhot, China) were obtained from local supermarkets in Beijing, China, and verified to be free of cells using microscopic methods. Somatic cell-spiked milk samples were pretreated by washing the solutions twice with Tris-HCl buffer (100× *g*, 10 min), followed by resuspension of the cells in 1 mL of Tris-HCl buffer. Each sample underwent three repetitions of testing and analysis.

### 2.9. Quantification of Somatic Cells in Real Milk Samples

**Figure 2:**
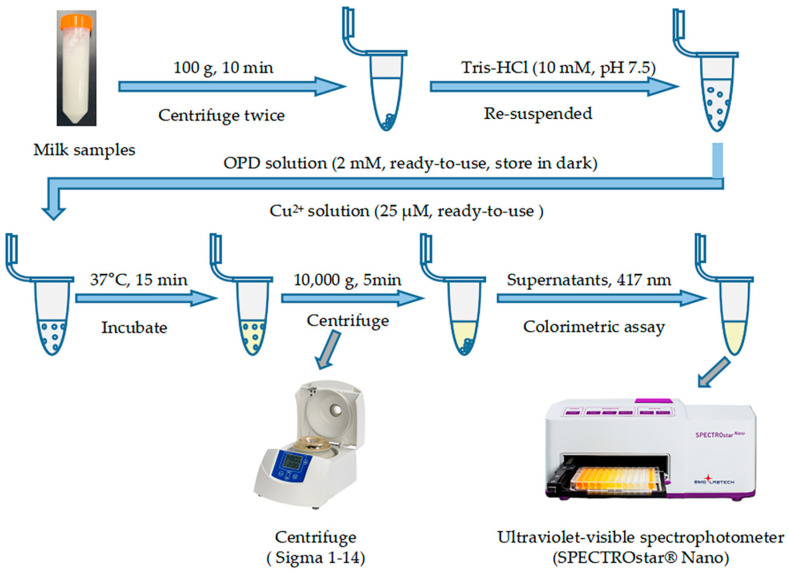
Flow chart of the OPD-Cu^2+^ system-based colorimetric method for SCC quantification.

Raw milk samples were obtained from a local dairy farm in Beijing, China. A volume of 1 mL of the raw milk was centrifuged twice (100× *g*, 10 min) and resuspended in Tris-HCl (10 mM). Cu^2+^ solution and OPD solution were then added to achieve final concentrations of 25 µM and 2 mM, respectively. The total reaction volume was 400 μL, and the mixture was incubated at 37 °C for 15 min in the dark. Following incubation, the samples were cooled to room temperature and centrifuged (10,000× *g*, 5 min). Absorbance signals of the resulting supernatants were measured at 417 nm. The detection method is outlined in Figure 2. Each sample was analyzed in triplicate, and results were compared with those obtained using the microscopic counting method.

### 2.10. Microscopic Counting

The traditional microscopic counting method was used to identify somatic cells in both the somatic cell-spiked and real milk samples. The samples were initially heated in a 40 °C water bath for 5 min, shaken thoroughly, and then allowed to cool. A 10 μL aliquot of the sample was spread on a slide treated with 95% ethanol. Once the samples were air-dried, the film on the slide was immersed in a modified Newman–Lampert stain solution for 15 min [33]. The sample was then washed and dried. Stained cells on the slides were subsequently counted using a microscope.

## 3. Results and Discussion

### 3.1. Feasibility of the OPD-Cu^2+^ System for the Detection of Somatic Cells

**Figure 3:**
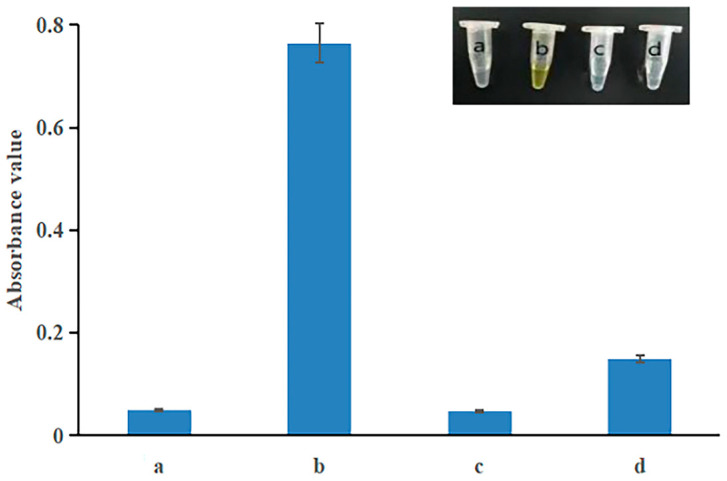
The somatic cells directly decreased the OPD-Cu^2+^ solution absorbance value. The absorbance values at 417 nm of OPD (**a**), OPD-Cu^2+^ (**b**), OPD–somatic cells (**c**), and OPD–Cu^2+^–somatic cells (**d**) were recorded. Insets: corresponding photos.

In this study, a colorimetric method based on the OPD-Cu^2+^ system was developed to accurately quantify somatic cells. As depicted in Figure 1, the method relies on the oxidation reaction between OPD and Cu^2+^, as well as the interaction between Cu^2+^ and somatic cells. Typically, the OPD solution is colorless and transparent, and no color change occurs when it is incubated for 2 h at 37 °C (Figure 3, tube a). However, the presence of Cu^2+^ induces the oxidation of OPD to OPDox. Following incubation, a change in the absorbance value of the solution was observed due to the formation of OPDox (Figure 3, tube b). Additionally, no signal was detected in the OPD–cell solutions without Cu^2+^ (Figure 3, tube c). Furthermore, the addition of somatic cells to the OPD-Cu^2+^ solution resulted in the inhibition of OPD oxidation by the cells interacting with Cu^2+^, leading to a reduction in the absorbance value (Figure 3, tube d). These findings indicate that somatic cells effectively consumed Cu^2+^ and competitively inhibited OPD oxidation. The observed decrease in absorbance value was thus related to the presence of somatic cells. Consequently, the OPD-Cu^2+^ system was utilized to establish a colorimetric method for somatic cell quantification, complementing the previously reported method for bacterial cell detection [34].

### 3.2. Optimization of the OPD-Cu^2+^ System Detection Conditions

#### 3.2.1. Optimization of the Cu^2+^ Concentration

**Figure 4:**
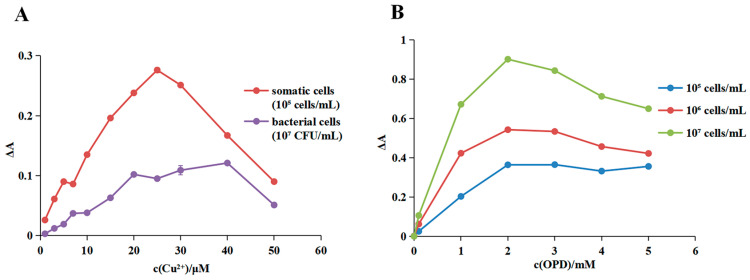
Optimization of the OPD-Cu^2+^ system detection conditions. (**A**) Optimization of Cu^2+^ concentration for somatic cell detection with the OPD-Cu^2+^ system. The optimal Cu^2+^ concentration was established by comparing the detection results of the somatic cell and bacterial samples. (**B**) Optimization of OPD concentration for somatic cell detection with the OPD-Cu^2+^ system. OPD was added to determine the concentration that produced the strongest signal. The optimized OPD concentration was subsequently validated with various concentrations of cell samples.

To determine the optimal Cu^2+^ concentration, the somatic cell and bacterial samples were analyzed, and the signal changes were compared across different Cu^2+^ concentrations. As illustrated in Figure 4A, for somatic cell detection, absorbance values varied with increasing Cu^2+^ concentrations, with the highest signals recorded at 25 μM Cu^2+^. Similarly, when the same method was applied to bacterial detection, the maximum signal change occurred at a Cu^2+^ concentration of 40 μM. These findings indicated that both somatic cells and bacteria reduced the colorimetric response of the OPD-Cu^2+^ system. Furthermore, somatic cells consumed more Cu^2+^ at the same concentration compared to bacterial cells, which may be attributed to their larger cellular volume or enzyme content [35]. The greatest difference in signal response between somatic cells and bacteria was observed at 25 μM Cu^2+^, suggesting minimal bacterial interference with somatic cell detection. Consequently, a Cu^2+^ concentration of 25 μM was selected for somatic cell detection.

#### 3.2.2. Optimization of the OPD Concentration

To improve the somatic cell detection signals, the signal variations of OPD–Cu^2+^–somatic cell solutions were analyzed at different OPD concentrations. Absorbance values of the detection system varied with increasing OPD concentration, with the highest signals recorded at 2 mM OPD (Figure 4B). Additionally, the same trend was observed with different concentrations of somatic cells in the mixture. These results confirmed that somatic cells reduced the colorimetric response of the OPD-Cu^2+^ system. Consequently, a concentration of 2 mM OPD was selected for constructing the somatic cell assay.

#### 3.2.3. Optimization of the Incubation Time

To enhance detection efficiency, the incubation time for the OPD-Cu^2+^ system-based colorimetric method was optimized for somatic cell detection. Somatic cells were subjected to incubation in the detection system and sampled every 15 min. As indicated in Table 2, within the concentration range of 10^4^ cells/mL to 10^6^ cells/mL, a linear relationship between absorbance signals and cell concentrations was observed, with a satisfactory correlation coefficient after 15 min of incubation. Thus, the optimal incubation time for this method was established as 15 min.

### 3.3. Somatic Cell Detection with the OPD-Cu^2+^ System 

**Figure 5:**
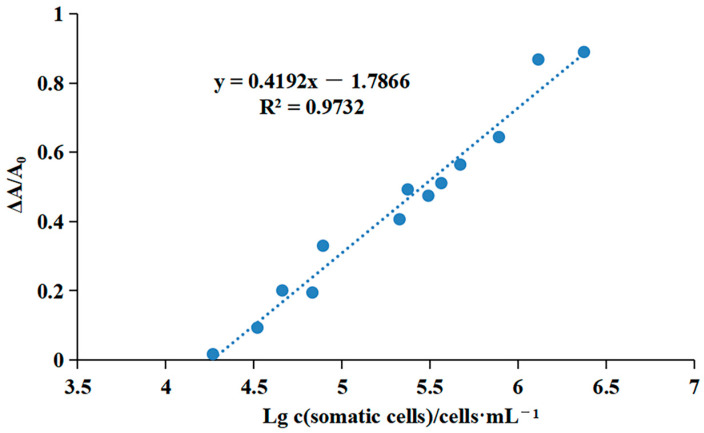
Standard curve for measuring SCC via the OPD-Cu^2+^ system. ΔA—the change in the absorbance signal between the blank control and the cell samples. A_0_—the absorbance signal of the blank control.

Following the optimization described in Section 3.2, somatic cells were quantified using the OPD-Cu^2+^ system, with an OPD concentration of 2 mM, a Cu^2+^ concentration of 25 μM, and an incubation time of 15 min. Various concentrations of somatic cells (10^4^ cells/mL to 10^6^ cells/mL) were introduced into the system, and absorbance signals were recorded. As depicted in Figure 5 a linear relationship was observed between the relative change in absorbance values and the logarithm of the somatic cell concentration. The mathematical formula for quantifying the somatic cell concentration (SCC) is y = 0.4192x − 1.7866, where y represents the relative change in absorbance signal (ΔA/A_0_) and x denotes the logarithm of the somatic cell concentration. These findings demonstrate that the developed method accurately quantifies somatic cells. Additionally, the detection limit of the somatic cell quantification method was found to be 1.85 × 10^4^ cells/mL. Given that the SCC in raw milk typically ranges from 2 × 10^4^ cells/mL to 2 × 10^5^ cells/mL [36], this method’s detection limit is adequate for its intended application. Consequently, the method is suitable for practical use in detecting somatic cells in raw milk. Furthermore, since the absorbance values are linearly related to the logarithm of the SCC, meticulous sample handling and accurate signal measurement are essential.

### 3.4. The Anti-Interference Ability of the OPD-Cu^2+^ Detection System

To determine if bacteria affect the OPD-Cu^2+^ system’s ability to detect somatic cells, signal changes in the presence of bacteria were assessed using the developed method. *P. fluorescens*, *A. baumannii*, and *L. lactis*, which are commonly found in raw milk [37,38,39,40,41,42], were used to simulate bacterial contamination in the anti-interference experiment. Bacteria at a concentration of 2 × 10^6^ CFU/mL were mixed with varying concentrations of somatic cells (10^4^ cells/mL to 10^6^ cells/mL) and introduced into the OPD-Cu^2+^ solution. The resulting signals were compared to those of the negative control (somatic cell detection alone). As shown in Table 3, no significant difference (α = 0.05, *p* = 0.97 > 0.05) was observed when bacteria were added to the somatic cell detection system. Furthermore, since the total bacterial count in raw milk typically ranges from 10^4^ CFU/mL to 10^5^ CFU/mL [43], it is suggested that bacteria in raw milk do not interfere with somatic cell detection using the OPD-Cu^2+^ system. Therefore, the OPD-Cu^2+^ system demonstrated strong anti-interference capability for somatic cell detection in raw milk. Additionally, when the OPD-Cu^2+^ system’s detection conditions were optimized, the bacteria also showed a good response at lower Cu^2+^ concentrations. Detection signals exhibited a linear relationship with bacterial concentration (Figures S1 and S2), consistent with previous research [25]. These findings indicate that this method could be developed as a novel approach for the simultaneous detection of somatic and bacterial cells in raw milk with varying Cu^2+^ concentrations.

### 3.5. Detection of Somatic Cells in the Artificially Contaminated Samples 

**Figure 6:**
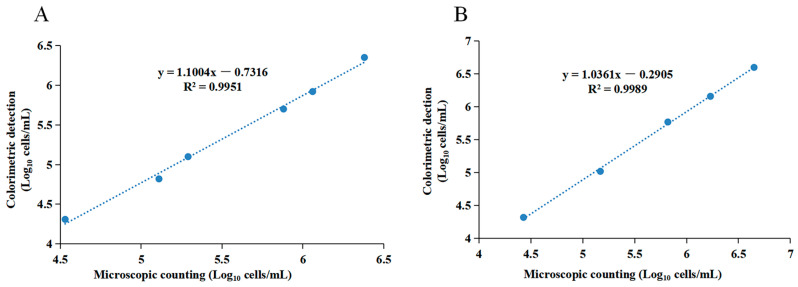
Quantification capability of the OPD-Cu^2+^ system-based colorimetric method in milk samples. (**A**) Relationship between the colorimetric detection and microscopic counting methods for the quantification of somatic cells in Tris-HCl. (**B**) Relationship between colorimetric detection and microscopic counting methods for the quantification of somatic cells in artificially contaminated milk samples.

To evaluate the reproducibility of the developed method, somatic cells (~10^6^ cells/mL) were spiked into five independent milk samples and quantified using the OPD-Cu^2+^ system. As presented in Table 4, the relative standard deviation (RSD) of the somatic cell quantification method was 2.1% in milk, demonstrating excellent reproducibility. Additionally, the recovery rate of somatic cells in milk was 88.9% with this method. Somatic cells were also detected using a microscopic method, and results were compared with those from the colorimetric method. Whole-fat sterilized milk samples were spiked with somatic cells (ranging from 10^4^ cells/mL to 10^6^ cells/mL) for evaluation, with somatic cells in Tris-HCl serving as controls. As shown in Figure 6A,B, a strong correlation was observed between results from the developed method and the microscopic counting method for somatic cell concentrations above 10^4^ cells/mL, with R^2^ = 0.9951 in Tris-HCl and R^2^ = 0.9989 in milk. These results indicate that the developed method accurately quantifies the somatic cell concentration in milk samples, and the detection range aligns with that of somatic cell concentrations in real samples [36].

### 3.6. Detection of Somatic Cells in Real Milk Samples by the Developed Method 

To assess whether the developed method accurately quantifies SCC in real samples, somatic cells in raw milk were detected following pretreatment. As indicated in Table 5, the method accurately quantified the SCC in raw milk, with results aligning with those obtained by the microscopic counting method (α = 0.05, *p* = 0.88 > 0.05). The detection results suggest that the developed method has fewer errors and provides more accurate results compared to the microscopic counting method. Therefore, the method is suitable for detecting somatic cells in real samples. Additionally, the SCC concentration in milk typically ranges from 2 × 10^4^ cells/mL to 2 × 10^5^ cells/mL [36], while the SCC concentration quantified in this study was about 2 × 10^4^ cells/mL. Consequently, the real sample results indicate that the quality of the raw milk collected was good and that the mammary glands of the sample cows were in good health.

### 3.7. Principle behind the OPD-Cu^2+^ System in Detecting Somatic Cells

In this research, it was observed that the introduction of somatic cells competitively inhibited the OPD oxidation reaction, resulting in reduced OPDox production and decreased absorbance signals. Somatic cells may deplete Cu^2+^ through two mechanisms: various enzymes in somatic cells reduce Cu^2+^ to Cu^+^ via redox reactions, and nonspecific binding of Cu^2+^ by the negatively charged cell surface. To investigate the response mechanism between somatic cells and Cu^2+^, CCCP, which inhibits cellular redox reactions [44], and negatively charged magnetic beads were added to the reaction system. As shown in Figure S3, the addition of CCCP led to a decrease in the signals of the OPD–Cu^2+^–somatic cell solution, with no significant differences observed compared to the system without CCCP. This indicates that Cu^2+^ consumption by somatic cells is not dependent on cellular redox reactions, differing from the detection mechanism employed for bacterial cell quantification [25]. Similarly, the addition of negatively charged beads did not result in significant changes in the absorbance values of the OPD–Cu^2+^–somatic cell solution. These results suggest that Cu^2+^ is not consumed by somatic cells through charge adsorption on the cell surface. Thus, somatic cells may deplete Cu^2+^ via a novel mechanism distinct from that of bacterial cells, warranting further investigation through more sophisticated experimental designs.

## 4. Conclusions

This study presents an OPD-Cu^2+^ system-based colorimetric method for the quantification of somatic cells in raw milk within 45 min. Changes in absorbance signals are linearly correlated with the logarithm of somatic cell concentration over the range of 10^4^ to 10^6^ cells/mL. The method’s advantages include its low cost and ease of use for somatic cell detection; however, the need for a centrifuge and a spectrophotometer may restrict its broader application. Additionally, the method demonstrated a notable benefit in accurately quantifying somatic cells in the presence of bacteria when assessing somatic cell counts in milk. The method effectively quantified somatic cells in real samples, making it suitable for practical use. In conclusion, this study introduces a novel method for detecting somatic cells, which is crucial for assessing raw milk quality and diagnosing the mammary gland health of dairy cows. Furthermore, the results confirmed that bacterial cells also exhibit a linear response to the OPD-Cu^2+^ system. These findings suggest that the colorimetric method holds potential for simultaneously detecting bacteria and somatic cells in raw milk with varying pretreatment procedures and Cu^2+^ concentrations. The method may also be applicable to detecting somatic cells in other food or biological samples. However, the reaction mechanism between somatic cells and Cu^2+^ remains unclear and warrants further investigation.

## Figures and Tables

**Table 1:** Common methods for detecting somatic cell count in raw milk.

Methods		Applicability	Advantages	Disadvantages
Direct detection methods	Fluorescence photoelectric counting	Enumeration method	Efficient operation, rapid detection, high reproducibility	Costly instruments and necessitates regular calibration; needs expertise of specialized personnel
	Coulter counting	Enumeration method	Rapid detection	Complex pretreatment
	Flow cytometry	Enumeration method	Straightforward and efficient detection	High cost
Indirect detection methods	California Mastitis Test (CMT)	Screening method	Low cost and easy to operate	Only quantifies the relative count of somatic cells; influenced by the subjective evaluations
	ATP bioluminescence detection	Screening method	Simple operation and rapid detection	Detection accuracy is low; easily interfered by bacteria
	Conductivity and pH detection	Screening method	Simpledetection process	Lacks accuracy, and the accurate results can only be achieved with a microscope

**Table 2:** Standard curves of somatic cell detection with different incubation times with the OPD-Cu^2+^ system.

Incubation Time	The Fitted Linear Equation	The Coefficient of Determination (R^2^)
0 min	/	/
15 min	y = 0.4075x − 1.7543	0.9875
30 min	y = 0.5791x − 2.7225	0.9426
45 min	y = 0.3995x − 1.7551	0.9437
60 min	y = 0.2668x − 0.9961	0.9087
75 min	y = 0.38x − 1.6469	0.9264
90 min	y = 0.2995x − 1.1134	0.9349
105 min	y = 0.3925x − 1.6689	0.9801
120 min	y = 0.293x − 1.1239	0.9278
135 min	y = 0.3944x − 1.6529	0.9827
150 min	y = 0.2946x − 1.1473	0.9213
165 min	y = 0.3834x − 1.5887	0.9867
180 min	y = 0.2887x − 1.074	0.9591

**Table 3:** The OPD-Cu^2+^ system exhibited strong anti-interference capability.

Concentration of Somatic Cells (Log 10)	The Change in Absorbance Value (ΔA)			
	Absence of Bacteria	RSD (%)	Presence of Bacteria	RSD (%)
4.03	0.003	19.2	0.002	18.9
5.38	0.048	1.20	0.048	2.08
5.73	0.074	2.70	0.076	1.32
5.88	0.099	1.54	0.102	1.96
5.96	0.126	1.59	0.127	1.57

**Table 4:** Reproducibility test for the analysis of milk spiked with somatic cells. Five independent samples were pretreated, and each sample was analyzed in three replicates.

Samples	Detection Result (*n* = 3)	
	Concentration of Somatic Cells (×10^6^ Cells/mL)	RSD (%)
1	1.93	0.75
2	1.98	0.75
3	1.91	0.43
4	2.01	1.14
5	1.93	0.43
Average	1.95	2.10

**Table 5:** Detection results of somatic cells in real samples. The OPD-Cu^2+^ method accurately quantified somatic cells in real samples.

Real Milk Samples	Colorimetric Detection (*n* = 3)		Microscopic Counting (*n* = 3)	
	Average (Cells/mL)	RSD (%)	Average (Cells/mL)	RSD (%)
1	1.35 × 10^4^	3.34	1.5 × 10^4^	16.7
2	2.83 × 10^4^	4.42	2.5 × 10^4^	10
3	1.92 × 10^4^	5.21	1.75 × 10^4^	20
4	1.19 × 10^4^	5.88	1.25 × 10^4^	14.3

## Data Availability

The original contributions presented in the study are included in the article/Supplementary Material, further inquiries can be directed to the corresponding author.

## Supplementary Materials

The following supporting information can be downloaded at: https://www.mdpi.com/article/10.3390/foods13182890/s1, Figure S1: OPD-Cu^2+^ system for the universal detection of common bacteria in raw milk; Figure S2: The standard curves of bacteria detection using the OPD-Cu^2+^ system-based colorimetric method; Figure S3: Investigation of the principle of the OPD-Cu^2+^ system for the detection of somatic cells.
