# Supplementary material for: The Accurate and Exclusive Quantification of Somatic Cells in Raw Milk with an OPD-Cu2+ System-Based Colorimetric Method
Source: Foods. 2024 Sep 12;13(18):2890. doi: 10.3390/foods13182890 (PMC11431095; doi:10.3390/foods13182890)

Supplementary Material

Accurate quantification of somatic cells in raw milk with an OPD-Cu^{2+} system-based colorimetric method

Menghui Xie[1,2,†], Meng Wang[1,†], Siyuan Liu[1], Yingying Liu[1], Ziquan Wang[1], Guoping Zhou[2], Zhiwei Sui[1*]

1 Center for Advanced Measurement Science, National Institute of Metrology, Beijing, 100029, China; mengwang@nim.ac.cn (Meng Wang); liusy@nim.ac.cn (Siyuan Liu); liuyy@nim.ac.cn (Yingying Liu); wangzq@nim.ac.cn (Ziquan Wang)
2 School of Life Science and Technology, Wuhan Polytechnic University, Wuhan, 430023, China; xiemenghui2021@163.com (Menghui Xie); wjczgp@163.com (Guoping Zhou)
† These authors contributed equally to this work.
*** Correspondence: suizhiwei_2001@163.com; Tel.: (+86 10 64526364)**

Content

Figure S1. OPD-Cu^{2+} system for the universal detection of common bacteria in raw milk. The Cu^{2+} concentration was set to 5 μM to verify the capacity of the OPD-Cu^{2+} system for detecting bacteria. As depicted in the results, common bacteria in raw milk such as *Escherichia coli* (*E. coli*), *Staphylococcus aureus* (*S. aureus*), and *Lactococcus lactis* (*L. lactis*) responded to the system. When these bacteria were introduced into the OPD-Cu^{2+} system, they reduced the production of OPDox by consuming Cu^{2+}, lowering the absorbance of the solution.

Figure S2. The standard curves of bacteria detection with the OPD-Cu^{2+} system-based colorimetric method. The mixture of dominant bacteria in raw milk was subjected to gradient dilution and detected with the OPD-Cu^{2+} system-based colorimetric method. It was found that the relative logarithmic change in absorbance values was linearly related to the logarithm of the bacterial concentration. This result suggests that the method has the potential to be developed as a novel method for the detection of bacteria in raw milk at low Cu^{2+} concentrations.

Figure S3. Investigation of the principle of the OPD-Cu^{2+} system for the detection of somatic cells. Absorbance value at 417 nm of OPD-Cu^{2+} (a), OPD-Cu^{2+}-somatic cells (b), OPD-Cu^{2+}-somatic cells-CCCP (c), and OPD-Cu^{2+}-somatic cells-charged magnetic beads (d) were recorded. Somatic cells may consume Cu^{2+} following two ways: various enzymes in

somatic cells reduce Cu^{2+} to Cu^{+} through redox reactions; another is the nonspecific binding of Cu^{2+} by the cell surface negative charge. Therefore, CCCP, which inhibits the cellular redox reaction, and negatively charged magnetic beads were accordingly added to the reaction system to verify the response mechanism between somatic cells and Cu^{2+}, respectively.

Figure S1.

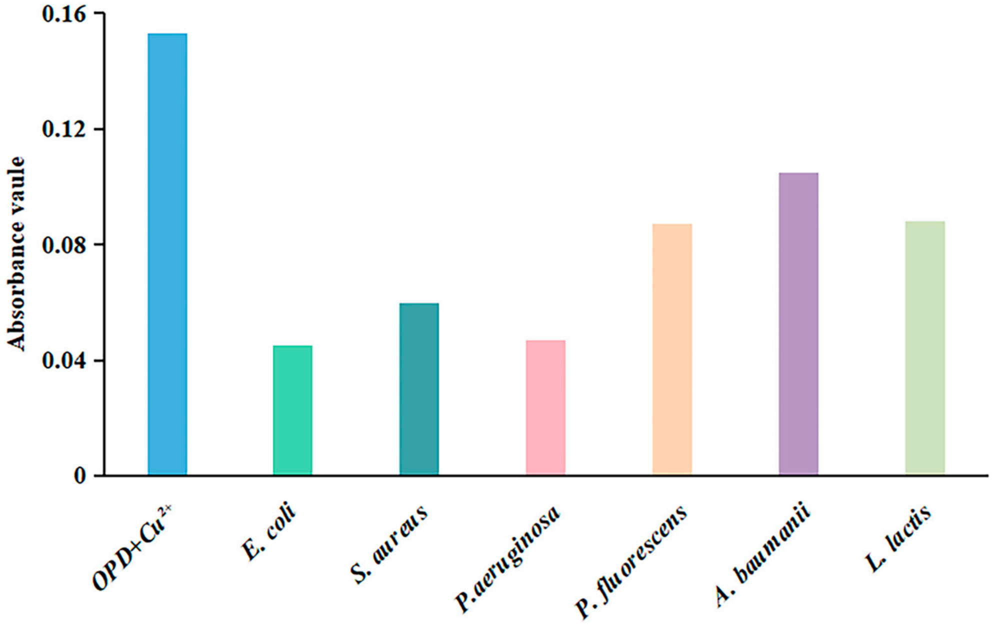
0.16
0.12
0.08
0.04
0
Absorbance vaule
OPD+Cu2+
E. coli
S. aureus
P.aeruginosa
P. fluorescens
A. baumanii
L. lactis

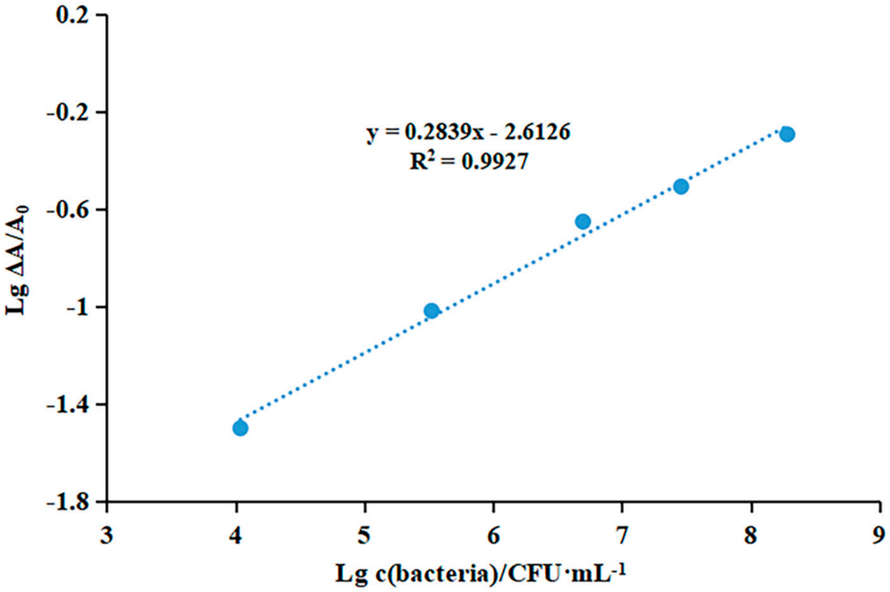
y = 0.2839x - 2.6126
R2 = 0.9927
Lg ΔA/A0
Lg c(bacteria)/CFU·mL-1
0.2
-0.2
-0.6
-1
-1.4
-1.8
3
4
5
6
7
8
9

Figure S2.

Figure S3.

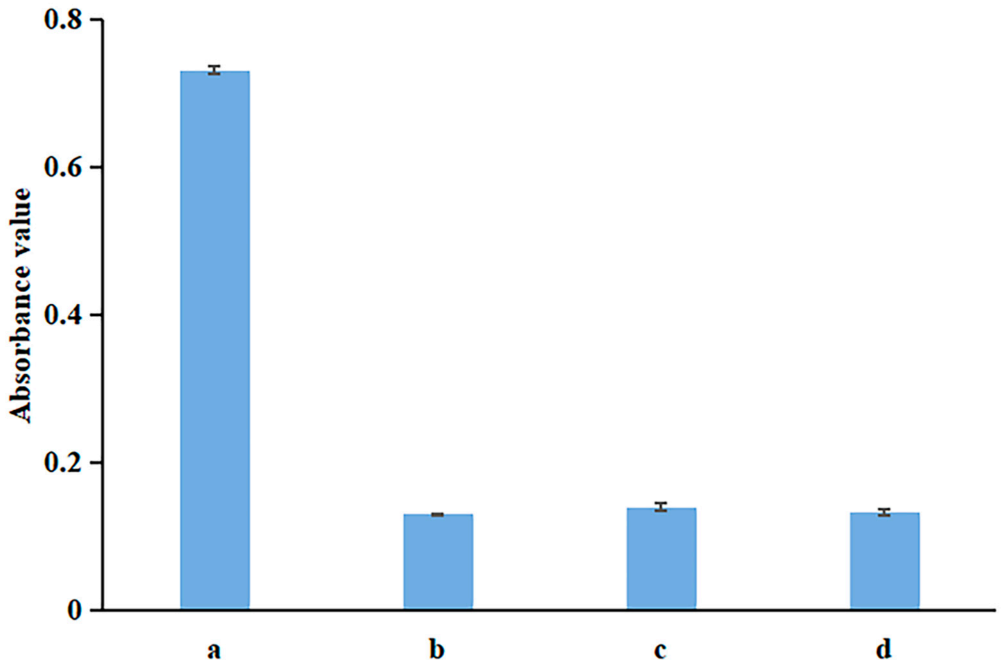

Supplement: Supplementary file 1 [file foods-13-02890-s001.zip › foods-3171340-supplementary.pdf]
